# Virulence Factors and Susceptibility to Ciprofloxacin, Vancomycin, Triclosan, and Chlorhexidine among Enterococci from Clinical Specimens, Food, and Wastewater

**DOI:** 10.3390/microorganisms12091808

**Published:** 2024-09-01

**Authors:** Diana Brlek Gorski, Josipa Vlainić, Ivana Škrlec, Silvia Novak, Željka Novosel, Zrinka Biloglav, Vanda Plečko, Ivan Kosalec

**Affiliations:** 1 Croatian Institute of Public Health, Rockefeller Str. 7, HR-10000 Zagreb, Croatia; diana.brlek-gorski@hzjz.hr; 2 Division of Molecular Medicine, Ruđer Bošković Institute, HR-10000 Zagreb, Croatia; josipa.vlainic@irb.hr; 3 Faculty of Dental Medicine and Health, Josip Juraj Strossmayer University of Osijek, HR-31000 Osijek, Croatia; iskrlec@fdmz.hr; 4 Department for Microbiology, Faculty of Pharmacy and Biochemistry, University of Zagreb, HR-10000 Zagreb, Croatia; 5 Department of Medical Statistics, Epidemiology and Medical Informatics, School of Public Health Andrija Štampar, HR-10000 Zagreb, Croatia; 6 School of Medicine, University of Zagreb, HR-10000 Zagreb, Croatia; 7 NHS Highland, Inverness IV2 3BW, UK; vanda.plecko2@nhs.scot

**Keywords:** *Enterococcus faecalis*, *Enterococcus faecium*, ciprofloxacin, vancomycin, chlorhexidine, triclosan, cell surface hydrophobicity, biofilm, hemolysis, antimicrobial resistance

## Abstract

*Enterococcus faecalis* and *E. faecium* are opportunistic pathogens commonly found in the microbiota of humans and other animals as well as in the environment. This article presents the results of antimicrobial susceptibility testing using phenotypic methods (broth microdilution and standardized disk diffusion) on selected clinical, food, and wastewater isolates of *E. faecalis* and *E. faecium.* The isolates were divided into subgroups based on their sensitivity to the following antibiotics: vancomycin (VAN) and ciprofloxacin (CIP), and biocides triclosan (TCL) and chlorhexidine (CHX). The study also investigated in vitro virulence factors, including biofilm formation ability, cell surface hydrophobicity (CSH) and β-hemolysis, to explore aspects of pathogenesis. In our study, regardless of the isolation source, VAN-resistant (VAN-R) and CIP-resistant (CIP-R) *E. faecalis* and *E. faecium* were detected. The highest proportion of CIP-R strains was found among clinical isolates of *E. faecalis* and *E. faecium*, with clinical *E. faecium* also showing the highest proportion of VAN-R strains. But the highest proportion of VAN-R *E. faecalis* strains was found in wastewater samples. The highest TCL MIC_90_ values for *E. faecalis* were found in wastewater isolates, while for *E. faecium,* the highest TCL MIC_90_ values were observed in food isolates. The highest CHX MIC_90_ values for both *E. faecalis* and *E. faecium* were identified in clinical specimens. The results obtained for *E. faecalis* did not indicate differences in TCL MIC and CHX MIC values with respect to sensitivity to VAN and CIP. Higher CHX MIC_50_ and CHX MIC_90_ values were obtained for CIP-R and VAN-R *E. faecium*. Among the tested isolates, 97.75% of the *E. faecalis* isolates produced biofilm, while 72.22% of the *E. faecium* isolates did so as well. In biofilm-forming strength categories III and IV, statistically significantly higher proportions of CIP-susceptible (CIP-S) and VAN-susceptible (VAN-S) *E. faecalis* were determined. In category III, there is no statistically significant difference in *E. faecium* CIP sensitivity. In category IV, we had a significantly higher proportion of CIP-R strains. On the other hand, the association between the moderate or strong category of biofilm formation and *E. faecium* VAN susceptibility was not significant. *E. faecalis* isolated from wastewater had a CSH index (HI) ≥ 50%, categorizing them as “moderate”, while all the other strains were categorized as “low” based on the CSH index. Among the *E. faecalis* isolates, cell surface hydrophobicity indices differed significantly across isolation sources. In contrast, *E. faecium* isolates showed similar hydrophobicity indices across isolation sources, with no significant difference found. Moreover, no correlation was found between the enterococcal cell surface hydrophobicity and biofilm formation in vitro. After anaerobic incubation, β-hemolytic activity was confirmed in 19.10% of the *E. faecalis* and 3.33% of the *E. faecium* strains.

## 1. Introduction

The *Enterococcus* genus consists of 63 child taxa, exhibiting diverse phenotypic characteristics [1]. Their isolation sources encompass both human and non-human environments, including animal hosts, plants, soil, water, and manufactured products. Enterococci are part of the normal microbiota of the gastrointestinal and biliary tracts in humans and animals [2,3]. However, these opportunistic pathogens can cause endocarditis, urinary, abdominal, pelvic, and other severe infections including line-related infections [4,5]. The most frequently isolated enterococci from patients with hospital-acquired infections (HAI) are *E. faecalis* and *E. faecium*, leading to significant mortality and morbidity [6].

According to the latest data from European Centre for Disease Prevention and Control (ECDC), there has been an increase in the number of reported isolates of *E. faecium* and *E. faecalis* resistant to one of the tested groups of antimicrobials (aminopenicillins, gentamicin (high-level resistance) and vancomycin), as well as an increasing number of hospital infections caused by enterococci. The ECDC highlights two major concerns related to resistant enterococci as follows: limited treatment options and epidemiological potential of spreading of resistant strains [6].

Antibiotic resistance and infections with resistant bacterial strains, including healthcare-associated infections, are rapidly increasing worldwide. The expansion of antibiotic resistance is mainly attributed to bacterial adaptation to various environmental conditions, such as the use of antibiotics in humans and in veterinary medicine, particularly in animal farming. Antibiotic-resistant strains represent the final outcome of a complex interaction of various mechanisms, whereas the hospital setting plays a crucial role in their development and spread [7,8,9]. Considering the use of large amounts of antibiotics and other antimicrobials (biocides), we can talk about antimicrobial resistance. The mentioned use of biocides calls into question the occurrence of cross-resistance. Sublethal exposure to antimicrobial agents can induce stress response systems in bacteria, potentially leading to the upregulation of genes that confer resistance to other antimicrobials. This adaptive response might involve changes in the cell wall structure or increased expression of efflux pumps [8,9].

Antibiotic-resistant strains of enterococci have been detected in various isolation sources [10]. Compared to streptococci and staphylococci, enterococci possess fewer virulence factors. However, their ability to adhere to tissues and other surfaces, to form biofilms, as well as their resistance to antibiotics makes them significant pathogens [11]. Antibiotic resistance in enterococci can be intrinsic (change in a genome and specifically related to e.g., cephalosporins) or acquired through mutation or the acquisition of new genetic elements (e.g., to vancomycin) [12].

Since enterococci are part of the normal intestinal microbiota, they are exposed to the antibiotics used in the treatment of various infections. Additionally, their ability to survive in the environment exposes them to the antibacterial agents used to disinfect surfaces, hands, and other areas. These are the conditions that can lead to a unique profile of virulence and antimicrobial resistance.

Cell surface hydrophobicity, an important virulence factor, modifies bacterial adhesion to surfaces and biofilm formation, particularly in infections such as endodontitis, endocarditis, and urinary tract infections [13,14]. The relationship between resistance to antibiotics and biocides and biofilm formation has been previously reported [15]. Hemolytic activity in Enterococcus strains makes them more harmful because breaking down red blood cells gives the bacteria nutrients and helps them avoid the host’s immune system. The hemolysis is caused by a toxin known as cytolysin [16]. Cytolysin exhibits β-hemolytic properties in humans and has bactericidal activity against other Gram-positive bacteria [5,17].

Biocides, including broad-spectrum disinfectants triclosan (TCL) and chlorhexidine (CHX), are commonly used against a wide range of Gram-positive and Gram-negative bacteria. However, some bacteria survive exposure to these agents and develop resistance or tolerance, potentially leading to cross-resistance to antibiotics [18,19]. Determining bacterial growth kinetics in the presence of low biocide concentrations can facilitate changes in bacterial phenotype [20,21,22]. Bacteria might modify their cell wall structure, enhance biofilm production, or activate stress response pathways. By monitoring these changes over time, researchers can gain insights into the adaptive strategies that bacteria use, which may lead to the development of more resistant strains. Schwaiger et al. presented the susceptibility of *E. faecium* and *E. faecalis* isolates from animals, food, and humans to the biocides, DDAC and formic acid, as well as to 28 antibiotics, both before and after laboratory adaptation [23]. Braoudaki et al. also reported differences in susceptibility to biocides and some antibiotics following the exposure of *Salmonella enterica* and *E. coli* O157 to subinhibitory concentrations of the biocides [24]. At bactericidal concentrations, these biocides are believed to act through multiple non-specific mechanisms, including damage to the cell membrane [25].

Deviations in the MIC values for TCL and CHX in *E. faecalis* and *E. faecium* have been reported, with the suggestion that if the MIC is increased (at least doubled) most microorganisms can be considered resistant [26]. Various factors affect MIC, such as toxicity, pharmacodynamics, concentration, and place and method of use. Determining MICs for a large number of isolates and biocides, following the standards set for antibiotics, helps to estimate epidemiological “cutoff” value (ECOFF). Morrissey et al. established ECOFF based on the normal MIC distribution from susceptibility testing of 53 *E. faecium* (32 mg/L) and 56 *E. faecalis* (64 mg/L) strains [27]. Analysis of the MIC values for TCL and CHX for *E. faecalis* and *E. faecium* revealed strains with increased MICs. It has been suggested if MIC is increased (at least doubled), most microorganisms can be considered resistant [26]. Considering that there are no clearly defined limits for biocide sensitivity, we use the limits set by individual laboratories based on MIC values. Since the concentration used is significantly higher than the MIC value, an increase in MIC should not be considered resistant but rather to have reduced sensitivity [26]. Since enterococcal susceptibility to biocides has an important role in infection control and prevention, the association between biocide resistance and cross-resistance to antibiotics has also been a topic of discussion [28]. The COVID-19 pandemic rapidly increased the use of biocides in various environments, including hospitals, food processing plants, households, and non-hospital settings, particularly specialized niches like sewer effluent [29].

This study aimed to determine the susceptibility of *E. faecalis* and *E. faecium* isolated from different sources (clinical specimens, food, and wastewater) to the antibiotics VAN and CIP and the biocides TCL and CHX. Additionally, we described and compared virulence factors, such as cell surface hydrophobicity and hemolysis and confirmed hypothesis that antibiotic resistance and low biocide susceptibility are associated with biofilm formation ability.

## 2. Materials and Methods

### 2.1. Collection of Strains

Tests were performed on 179 (N = 179) *E. faecalis* and *E. faecium* strains from the strain collection held at the Croatian Institute of Public Health, Food Microbiology Department. These strains were isolated from clinical and environmental sources (food, wastewater). Clinical specimens were obtained from samples collected as part of routine hospital work at University Hospital Centre Zagreb. These isolates were derived from blood cultures, urines, fecal samples, rectal swabs, perianal swabs, wound swabs, and skin swabs. Isolates from food and wastewater were collected during routine work at the Croatian Institute of Public Health, Food Microbiology Department. For the purpose of this study, isolates of clinical specimen *E. faecalis* (n = 29) and *E. faecium* (n = 30), from food *E. faecalis* (n = 30) and *E. faecium* (n = 30), and from wastewater *E. faecalis* (n = 30) and *E. faecium* (n = 30) were tested. After isolation and identification, the isolates were stored on the Microbank system (Pro-Lab Diagnostics, Richmond Hill, ON, Canada) in the vials containing 25 beads to prevent changes in characteristics and repetitive subculturing of the strains.

Ethical approvals were obtained from the University Hospital Centre Zagreb (No. 02/21-LJH), the Croatian National Institute of Public Health (No. 001-418/1-11), and the Faculty of Pharmacy and Biochemistry University of Zagreb (No. 251-62-03-12-26).

### 2.2. Isolation and Identification

Isolates were previously identified and stored on a Microbank system (Pro-Lab Diagnostics). Before starting the planned tests, a single bead was plated on selective solid media Kanamycin Aesculin Azide Agar (KEA) (Liofilchem, Teramo, Italy) and Tryptic Soy Agar (TSA), (Liofilchem, Italy). Incubation was carried out under aerobic conditions at 35–37 °C/24–48 h.

Following incubation, preliminary identification was performed based on morphological observation such as growth on bile esculin agar (round, white or grey colonies about 1–2 mm in diameter, surrounded by black zones), catalase activity (negative), Gram staining (single Gram-positive cocci, in pairs, or in short chains). The next identification step was carried out with the BBL Crystal GP ID panel (Becton, Dickinson and Company, Hackensack, NJ, USA). The test inoculum was prepared with the inoculum fluid, and the wells were filled. After incubation, the wells were examined for color changes or the presence of fluorescence that resulted from the metabolic activities of the microorganisms. Each sample was manually read on the Crystal panel viewer to obtain a profile number, and the results were interpreted with the Crystal MIND software (BD BBL Crystal MIND Software V5.05/Installer V1.5).

Species-level strain identification was carried out using Matrix-Assisted Laser Desorption/Ionization Time-Of-Flight Mass Spectrometry (MALDI-TOF MS Syrius) with MALDI Biotyper 3.0 software (Bruker Daltonics, Bremen, Germany). According to the manufacturer’s instructions, the direct colony method was applied. Colonies of enterococci were spotted on the MALDI target plate and then overlaid with 1 μL of HCCA (α-Cyano-4-hydroxycinnamic acid) matrix solution and air-dried. The plate was then placed in the instrument, and the spectrum was read and compared with the data stored in the instrument’s software (MALDI Biotyper software, MBT Compass HT software (version 5.1.300) and MBT reference library (version 12.0.0.0)). Identification was provided with score values and consistency categories (A–C). The meanings of the score values are as follows: range 2.00–3.00 indicates high confidence identification (green); range 1.70–1.99 indicates low confidence identification; range 0.00–1.69 indicates no organism identification possible. Calibration was performed using *E. coli* as the standard (BTS, Bruker Daltonics, Germany) and *E. faecalis* ATCC 29212 as the positive control. Each test was performed in triplicate [30,31].

### 2.3. Antimicrobial Susceptibility Testing

Susceptibility testing for *E. faecalis* and *E. faecium* to ciprofloxacin (CIP) and vancomycin (VAN) was performed at the Croatian National Institute of Public Health using the standardized disk diffusion method, interpreted in accordance with the European Committee on Antimicrobial Susceptibility Testing (EUCAST) guidelines [32]. Testing was conducted on Mueller–Hinton (MH) agar (Liofilchem, Italy). A 0.5 McFarland density inoculum was applied to the agar surface, and antibiotic discs of CIP (5 µg) and VAN (5 µg) (Mast Group Ltd., Merseyside, UK) were overlaid. The plates were incubated at 35 ± 1 °C for 24 h. Based on the measured diameter of the zone of inhibition around the antibiotic disc (in mm), the strains were classified as susceptible (S) or resistant (R) [33]. For strains that exhibited resistance using the disk diffusion method, the minimum inhibitory concentration (MIC) was determined using the ETEST strip (bioMérieux, Lyon, France), which was placed onto a plate inoculated with bacteria. The MIC was read from the scale following the incubation period. For quality control, *E. faecalis* ATCC 29212 was used. The ETEST was used as an additional confirmatory test for strains that showed resistance by disk diffusion, according to the EUCAST guidelines. For both *E. faecalis* and *E. faecium,* the MIC breakpoints for VAN and CIP were defined as susceptible (S) at ≤4 mg/L and resistant (R) at >4 mg/L (EUCAST breakpoint tables for interpretation of MICs and zone diameters Version 14.0, valid from 1 January 2024) [33].

### 2.4. Triclosan and Chlorhexidine Susceptibility Testing–Minimum Inhibitory Concentration

The susceptibility of enterococci to triclosan (TCL) and chlorhexidine (CHX) was determined by measuring the MIC using the microdilution method, following EN ISO 20776-1 [34]. Briefly, 100 µL of an inoculum containing 5 × 10^5^ CFU/mL was plated, with the expectation of observing 20–80 colonies after incubation. The concentrations tested ranged from 0.125 to 128 mg/L. Minimum bactericidal concentration (MBC) was performed by culturing 10 µL from each microtiter plate well on MH agar at 37 °C/48 h. MBC is defined as the lowest concentration at which no growth was observed on the MH agar (MBC data not included in this article). Results were presented as MIC_50_, indicating the concentration (mg/L) at which 50% or more of the tested strains were inhibited, and MIC_90_, indicating the concentration at which 90% or more of the tested strains were inhibited.

### 2.5. Biofilm Formation

The biofilm plate assay was conducted using Tryptic Soy Broth (TSB) (Liofilchem, Italy) supplemented with 1% *w*/*v* glucose and strains adjusted to 0.5 McFarland (10^8^ CFU/mL) in physiological saline. For each strain, a 20 µL suspension was inoculated in 180 µL of TSB supplemented with 1% glucose in sterile fat-bottomed microtiter plate. After 24 h of incubation at 37 °C, the medium was discarded, and the plates were washed with phosphate-buffered saline (PBS). This washing procedure was repeated three times to remove non-adherent cells. After overnight air drying, the plates were stained using 200 µL of 1% *w*/*w* crystal violet solution for 15 min, then washed under running tap water and dried. Ethanol (200 µL, 96% *v*/*v*) was added to each well, and the optical density (OD_570_) was measured using an absorbance microplate reader (Azure Biosystems, Dublin, CA, USA). All assays were performed in triplicate, and 200 µL of TSB supplemented with 1% glucose was used as a negative control [35].

The ability to form biofilm was classified as follows: no biofilm formation (I), weak (II), moderate (III), and strong (IV) [36].

### 2.6. Cell Surface Hydrophobicity

Enterococcal cultures were suspended in 0.9% w/v NaCl, and optical density (OD_600_) was measured (A_0_ = 0.8–1). Xylene (1.7 mL) was then added to the suspension and vortexed for 2 min. After allowing the mixture to separate at room temperature, the optical density of the aqueous phase was measured (A). Based on the affinity of the bacteria for xylene (hydrophobic solvent), cell surface hydrophobicity index (HI) was calculated as a numerical value representing a measure of how hydrophobic the bacterial cell surface is. HI (%) was calculated using the following equation: HI (%) = ((A_0_ − A)/A_0_) × 100 [36]. According to Tahmourespour et al., the classifications for hydrophobicity are as follows: highly hydrophobic (HI > 70%), moderate hydrophobic (HI 50–70%), and low hydrophobic (HI < 50%) groups [13]. Bacteria with a greater ability to adhere to xylene will exhibit a higher HI, indicating higher hydrophobicity.

### 2.7. Assessment of β-Hemolytic Activity

The hemolytic phenotype was determined using horse blood agar (bioMerieux, France). Following incubation (37 °C/24–48 h) under both aerobic and anaerobic (GENbag, bioMérieux) conditions, colonies with a clear zone around them were considered β-hemolytic positive. In contrast, the strains were interpreted as negative if they produced a greenish zone (α-hemolysis) or no zone at all. *S. aureus* ATCC 25923 was used as a reference strain (positive control) [37]. Additionally, we selected three strains with hemolytic properties at 37 °C and incubated them at the following different temperatures: 5 °C, 15 °C, 25 °C, and 35 °C. None of the selected isolates showed hemolysis at 5 °C and 15 °C, but hemolytic activity was observed at 25 °C and 35 °C. Minimal growth was observed at 5 °C. At 15 °C, some limited growth was observed but it was less robust compared to growth at the higher temperatures. The reduced growth at the lower temperatures could explain the absence of hemolysis at these temperatures.

### 2.8. Statistical Analysis

Descriptive and analytical statistical methods were used, and the results were presented numerically and graphically. The Shapiro–Wilk test was used to assess the normality of distribution. Continuous data were analyzed with a *t*-test or Mann–Whitney U test. Spearman’s correlation and the chi-squared test were used to measure the strength and direction of the association between biofilm-formation ability and susceptibility to biocides and antibiotics. ANOVA or the Kruskal–Wallis test were used to compare the means of more than two groups.

For analysis, strains were dichotomized according to biofilm formation ability as no biofilm producers (I) and biofilm producers (II, III, IV). MIC was also dichotomized into two categories as follow: the first included median and values below, and the second included values above the median. The level of statistical significance was set at 0.05. The analyses were performed using SPSS, version 27 (IBM, Armonk, NY, USA), and the JASP program [38].

## 3. Results

### 3.1. Antimicrobial Susceptibility of Enterococcus *spp*. to Vancomycin and Ciprofloxacin according to Origin

The data indicate that there was no statistically significant difference in the distribution of CIP-resistant (CIP-R) and CIP-susceptible (CIP-S) *E. faecalis* according to the isolation site, as the *p*-values for both CIP-R and CIP-S were greater than 0.05 (0.094 and 0.368, respectively). The distribution of VAN-resistant (VAN-R) and VAN-susceptible (VAN-S) *E. faecalis* for clinical, food, and wastewater samples also did not show significant difference. *E. faecalis* had the highest proportion of CIP-R strains in a group of clinical specimens (54.55%), while the highest proportion of VAN-R strains was observed in wastewater (63.64%). Overall, the sensitive and resistant strains were similarly distributed across various isolation sites. Although the *p*-value for VAN-R *E. faecalis* (0.078) suggests a trend towards significance, it still does not reach the threshold for statistical significance (0.05). The *p*-value for VAN-S *E. faecalis* (0.707) clearly indicates no significant difference across isolation sites.

In contrast, the distribution of CIP-R *E. faecium* across clinical, food, and wastewater samples were statistically significant (*p* = 0.005). The clinical samples exhibited a significantly higher percentage of resistant samples compared to the food and wastewater samples. The distribution of CIP-S *E. faecium* strains across these isolation sites was not significantly different (*p* = 0.493). The clinical isolates also showed a significantly higher percentage of VAN-R samples compared to the food and wastewater isolates, with the distribution of VAN-R *E. faecium* being statistically significant (*p* < 0.001). Furthermore, the distribution of VAN-S *E. faecium* was statistically significant (*p* < 0.001), with food and wastewater isolates showing significantly higher percentages of susceptible strains compared to the clinical ones (Table 1).

### 3.2. Antimicrobial Susceptibility of Enterococcus *spp*. to Triclosan and Chlorhexidine

The TCL MIC_50_ values for *E. faecalis* were from 8 mg/L to 16 mg/L, while the MIC_90_ were from 8 mg/L to 32 mg/L. For *E. faecium*, the TCL MIC_50_ values ranged from 4 mg/L to 8 mg/L, and MIC_90_ values ranged from 8 mg/L to 16 mg/L. The CHX MIC_50_ values for *E. faecalis* ranged from 4 mg/L to 16 mg/L, with MIC_90_ ranging from 8 mg/L to 32 mg/L. For *E. faecium*, the CHX MIC_50_ values ranged from 2 mg/L to 8 mg/L and the MIC_90_ from 4 mg/L to 16 mg/L.

The highest TCL MIC_90_ values for *E. faecalis* were found in the wastewater isolates, while for *E. faecium,* the highest TCL MIC_90_ values were observed in the food isolates. The highest CHX MIC_90_ values for both *E. faecalis* and *E. faecium* were identified in the clinical specimens (Table 2).

The relationship between the MIC values for triclosan and chlorhexidine, and the results for vancomycin and ciprofloxacin is shown in Supplementary Table S1 for *E. faecium* and in Supplementary Table S2 for *E. faecalis.*

Data in Supplementary Table S1 show that CIP-R and VAN-R *E. faecium* isolates generally had higher sensitivity to TCL compared to their sensitive counterparts. However, higher CHX MIC_50_ and CHX MIC_90_ values were obtained for CIP-R and VAN-R *E. faecium*, which could indicate lower sensitivity to CHX among CIP- and VAN-resistant isolates.

CIP-R and CIP-S *E. faecalis* had similar MIC_50_ and MIC_90_ values for both TCL and CHX. The same was true for VAN-R and VAN-S *E. faecalis* for TCL MIC_50_, TCL MIC_90_, and CHX MIC_50_, with the difference being that CHX MIC_90_ increased twofold in VAN-S strains. The results obtained for *E. faecalis* did not indicate differences in the TCL MIC and CHX MIC values with respect to sensitivity to VAN and CIP (Supplementary Table S2).

### 3.3. Biofilm Formation Ability

Among the *E. faecalis* isolates, 97.75% (n = 87) produced biofilm; 93.26% (n = 83) produced moderate (III) and strong (IV) biofilms and 4.49% (n = 4) produced weak (II) biofilms. Only 2.25% (n = 2) strains could not produce biofilm. The *E. faecium* isolates produced biofilms in 72.22% (n = 65) of samples, whereas 65.56% (n = 59) produced moderate (III) and strong (IV) biofilms and 6.67% (n = 6) produced weak (II) biofilms. Still, 27.78% (N = 25) *E. faecium* isolates could not produce biofilm.

Among *E. faecalis*, biofilm producers were from the food isolates (n = 29) or clinical specimens (n = 30) and 93.33% (N = 28) were from the wastewater isolates. The *E. faecium* biofilm producers were in 86.67% (n = 26) of the food isolates, in 96.67% (n = 29) of the clinical specimens and 33.33% (n = 10) of the wastewater isolates. Distribution of the biofilm-forming strength of *E. faecalis* and *E. faecium* isolated from different sources is presented in Table 3.

#### 3.3.1. *E. faecalis*—Association between Biofilm-Forming Strength and CIP and VAN Susceptibility

In weak category (II), all the strains were susceptible to CIP and VAN. The association was significant between CIP and VAN susceptibility and biofilm formation in moderate (III) and strong categories (IV). In categories III and IV, statistically significantly higher proportions of *E. faecalis* sensitive to CIP were determined (*p* = 0.009 and <0.001). The same applies to *E. faecalis* sensitive to VAN (*p* = 0.008 and <0.001) (Table 4).

#### 3.3.2. *E. faecium*—Association between Biofilm-Forming Capacity and CIP and VAN Susceptibility

In category III, there is no statistically significant difference in *E. faecium* susceptibility to CIP (*p* = 0.166). In category IV, the difference is statistically significant (*p* = 0.018), and thus we statistically determined a significantly higher proportion of the strains resistant to CIP in the category of strong biofilm producers. On the other hand, the association between the moderate or strong categories of biofilm formation and *E. faecium* VAN susceptibility was not significant (*p* = 0.166 and *p* = 0.238) (Table 5).

#### 3.3.3. *E. faecalis*—Association between Biofilm-Forming Strength and CHX and TCL MICs

For isolates of *E. faecalis,* the ability to form biofilms (in II, III, and IV category, N = 87) and the MIC for CHX (values above or below median) were not associated (r= −0.044, *p* = 0.686); as opposed to the MIC for TCL, where the association reached statistical significance (r = −0.261, *p* = 0.014). In addition, the increase in MIC values for both disinfectants tested was positively correlated with the decreased biofilm production.

#### 3.3.4. *E. faecium*—Association between Biofilm-Forming Strength and CHX and TCL MICs

The *E. faecium* biofilm formation ability in category II, III, and IV (N = 65) was not associated with MIC value for CHX (r = 0.160, n = 65, *p* = 0.202), but its increase was associated with increased biofilm production. Similarly, the MIC values for TCL and the ability to form biofilms for all the categories of biofilm production (r = −0.037, *p* = 0.768), with an increase in the MIC values associated with a decrease in biofilm production.

### 3.4. Cell Surface Hydrophobicity Index

**Figure 1:**
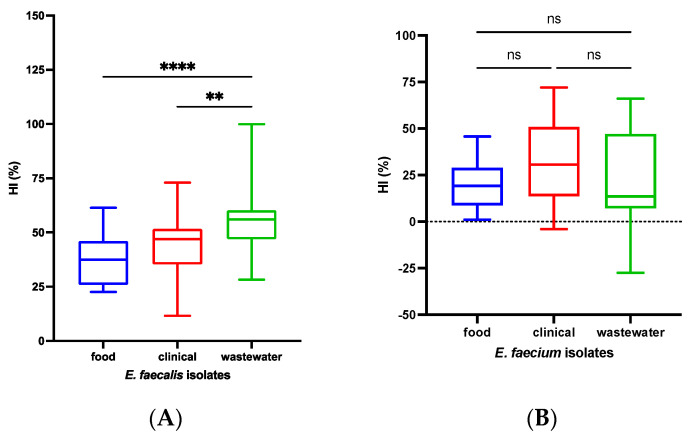
Cell surface hydrophobicity indices of *E. faecalis* (**A**) and *E. faecium* (**B**) isolates; ** *p* < 0.001; **** *p* < 0.0001; ns = not significant.

The measured cell surface hydrophobicity indices HI (%) for *E. faecalis* were as follows: food isolates had an HI of 34.70%, clinical specimens had an HI of 42.21%, and wastewater isolates had an HI of 52.15%. For *E. faecium,* the HI values were 17.9% for food, 29.14% for clinical specimens, and 23.50% for wastewater isolates. Based on the obtained hydrophobicity indices, all the *E. faecalis* and *E. faecium* isolates were classified as “low”, with the exception of the *E. faecalis* isolates from wastewater, which were classified as “moderate” (Figure 1).

Among *E. faecalis* isolates, cell surface hydrophobicity indices differed significantly across isolation sources (*p* < 0.001). Food and clinical specimens had similar indices (*p* = 0.08), whereas significant differences were observed between the food and wastewater isolates (** *p* < 0.001) and between the clinical and wastewater isolates (**** *p* < 0.0001). In contrast, the *E. faecium* isolates showed similar hydrophobicity indices across isolation sources, with no significant difference found (*p* = 0.13).

### 3.5. Hemolytic Activity

The hemolytic activity after aerobic and anaerobic incubation on horse blood agar of *E. faecalis* and *E. faecium* isolated from different sources is presented in Table 6. Regardless of the origin of the strain, β-hemolytic activity was confirmed for 13.48% (12/89) of *E. faecalis* strains under aerobic conditions and for 17.98% (16/89) of strains under anaerobic conditions (Table 6). For the *E. faecium* strains after aerobic and anaerobic incubation, none of the clinical isolates and wastewater strains showed hemolysis, and 10.00% of food isolates (N = 3) had a β-hemolytic zone under both aerobic and anaerobic conditions.

## 4. Discussion

*E. faecalis* and *E. faecium* contribute to the burden of nosocomial infections worldwide and have major clinical relevance. Additionally, enterococci can persist in the environment, particularly in the presence of organic material, and their persistence can be associated with reduced susceptibility to antibiotics and biocides.

Based on the susceptibility tests presented in this study, it can be concluded that the origin of a strain is associated with its susceptibility pattern, aligning with the One Health concept [39]. Antimicrobial resistance (AMR), especially resistance to vancomycin (VAN), which is a last-line treatment for Gram-positive hospital infections, is particularly concerning. The World Health Organization (WHO) considers VAN-resistant *E. faecium* a high-priority pathogen [40,41]. It should be emphasized that *E. faecium*, *Staphylococcus aureus*, *Klebsiella pneumoniae*, *Acinetobacter baumannii*, *Pseudomonas aeruginosa*, and *Enterobacter* spp. belong to the ESKAPE group of multidrug-resistant opportunistic pathogens [41].

There is an increasing trend of *E. faecalis* resistance to fluoroquinolones [42,43]. CIP resistance genes from enterococci can be transferred to other gut bacteria, and this transfer pathway highlights the importance of comprehensive surveillance and control measures. In our study, the data show no statistically significant difference in the distribution of CIP-R and CIP-S *E. faecalis* based on the isolation site, nor for VAN-R and VAN-S *E. faecalis*. The highest proportion of CIP-R *E. faecalis* strains was found in clinical specimens, while VAN-R strains were most prevalent in wastewater. For *E. faecium*, the distribution of CIP-R strains across clinical, food, and wastewater samples was statistically significant, with clinical samples showing a higher percentage of resistant strains. The distribution of CIP-S *E. faecium* across these sites was not significantly different. The clinical isolates also had a significantly higher percentage of VAN-R *E. faecium* compared to the food and wastewater isolates, with the distribution of both VAN-R and VAN-S *E. faecium* being statistically significant. In the study by Sobhanipoor et al., the results also demonstrated a higher proportion of clinical isolates (70%) compared to non-clinical isolates (52%) of enterococci [44].

Moreover, the inappropriate use of antibiotics and biocides, such as insufficient treatment duration and suboptimal concentration, can lead to selective pressure, reduced biocide efficacy, and the development of bacterial resistance [45]. In the case of enterococci, increased biocide tolerance is attributed to stress-induced protective mechanisms triggered by sublethal concentrations resulting from improper use or residues remaining in the environment [46].

The MIC levels for TCL and CHX as a measure of biocide effectiveness were also assessed. Among the tested strains, the highest MIC values were observed for CHX. For TCL, the highest MIC values were found in the *E. faecalis* isolates from wastewater. The interpretation of these results is limited since the strict criteria for MIC levels for biocides are not well defined. Still, to provide some estimates, researchers have used the average MIC values; the MIC values up to 2-fold cannot be considered as resistance but as decreased susceptibility or tolerance instead [47]. Thus, these results suggest that the MIC values for both disinfectants vary depending on the isolation site and does not indicate reduced susceptibility/tolerance. Exposure to TCL occurs primarily through consumer products, such as soaps, toothpaste, cosmetics, but this biocide is also a common pollutant in wastewater [48]. Its widespread use could lead to the development of TCL-resistant bacteria in the environment [47].

Studies have shown that CHX has higher activity compared to other biocides, including TCL [49]. CHX is a cationic antiseptic [50] used in various applications, from hand hygiene to antiseptic oral rinses [45]. Its increasing use, particularly against multidrug-resistant strains with varying sensitivities to CHX, underscores the importance of monitoring the evolution of *E. faecalis* tolerance [51]. Our study has shown that the highest CHX MIC_90_ values for both *E. faecalis* and *E. faecium* were identified in clinical specimens. However, higher CHX MIC_50_ and CHX MIC_90_ values were obtained for CIP-R and VAN-R *E. faecium*, which could indicate lower sensitivity to CHX among CIP- and VAN-resistant *E. faecium* isolates. One study demonstrated that a CHX MIC value greater than or equal to 64 mg/L is considered reduced susceptibility, and that 38% of non-clinical and 63% of clinical isolates show reduced susceptibility to CHX [44].

Previous studies of enterococci have reported similar or slightly higher MIC values than in this study, possibly due to differences in the isolation sites. Additionally, vancomycin-resistant enterococci (VRE) are less sensitive to CHX than VAN-susceptible ones [49]. Sobhanipoor et al. reported a CHX MIC_50_ of 64 mg/L and a CHX MIC_90_ of 128 mg/L for VAN-R enterococci, and a CHX MIC_50_ of 32 mg/L and a CHX MIC_90_ of 64 mg/L for VAN-S enterococci [44]. The same study noted that the CHX MIC results for clinical isolates of enterococci were higher than those reported in other studies, which may suggest a greater exposure to CHX in that region. According to Pereira et al., CHX MIC for clinical *E. faecalis* (4.8 mg/L) is higher than from the food isolates (4.1 mg/L) but similar to that of the environmental isolates (4.8 mg/L) [51]. Higher CHX MIC values in isolates from food and increasing CHX MIC values in isolates from human infections indicate the adaptability of the *E. faecalis* population in environments where CHX is intensively used [26]. The results obtained for *E. faecalis* did not indicate differences in TCL MIC and CHX MIC values with respect to sensitivity to VAN and CIP.

Considering that the range of MIC_50_ and MIC_90_ results for TCL and CHX did not suggest reduced susceptibility/tolerance, future studies should consider performing exposure test with subinhibitory doses of these biocides followed by repeat MIC testing. It is important to note that the MICs for TCL and CHX were not considered high enough in relation to their in-use concentrations.

Biofilm-forming ability is considered a significant virulence factor. Results from this study indicate that 97.75% of *E. faecalis* isolates and 72.22%*E. faecium* isolates formed biofilm, regardless of the isolation site (Table 3). The results align with findings from several studies that have reported 60 to 80% of clinical samples producing biofilm. The association between biofilm formation and susceptibility to CIP and VAN was evaluated. However, some studies have noted that only 49% VAN-R and 33% VAN-S isolates could produce biofilm [52]. In this study, in biofilm-forming strength categories III and IV, statistically significantly higher proportions of CIP-S and VAN-S *E. faecalis* were observed. Those results reject the hypothesis that a greater ability to form biofilms is associated with a higher proportion of CIP- and VAN-resistant *E. faecalis* strains. In category III, there was no statistically significant difference in CIP sensitivity among the *E. faecium* strains. However, in category IV, we observed a significantly higher proportion of CIP-R *E. faecium* strains. On the other hand, the association between moderate or strong biofilm formation and VAN susceptibility in *E. faecium* was not significant.

Hydrophobicity plays a crucial role in microbial infections, influencing bacterial adhesion to various abiotic and biotic surfaces and being associated with biofilm formation ability [53]. In our study, all strains were classified as “low”, except for the *E. faecalis* isolates from wastewater, which were classified as “moderate” (Figure 1). Significant differences in hydrophobicity indices were observed across the isolation sources for *E. faecalis* but not for *E. faecium*. The low hydrophobicity values and the proportion of biofilm-forming strains suggest that, as noted by Cho et al., there may be no direct association between hydrophobicity index (HI) and biofilm formation [54]. Similarly, Tahmourespur’s research indicated that antibiotic resistance and cell surface hydrophobicity did not show a linear correlation [13].

After anaerobic incubation, β-hemolytic activity was observed in 19.10% of *E. faecalis* and 3.33% *E. faecium* strains. These findings are consistent with Elsner et al.’s study, which reported β-hemolytic activity in 16% of *E. faecalis* and 0% of *E. faecium* isolates [55]. The observed differences in hemolytic activity may be attributed to variations in test conditions, such as the type of blood agar used, incubation conditions, and temperature. Our results indicate that more isolates exhibited hemolytic properties when incubated anaerobically (4.5%) regardless of the isolation site.

Infection models and epidemiologic studies have shown that enterococcal cytolysin, a hemolytic virulence factor, increases the virulence of enterococci and patient mortality [56]. Cytolysin is associated with increased severity of infection [57] as well as higher resistance to various antibiotics [56]. Cytolysin-mediated hemolysis occurs in certain erythrocytes, particularly those from humans, equines, bovines, and rabbits, but not in sheep and goat erythrocytes [56]. Our study’s results align with these findings, as *E. faecalis* from the clinical specimens, food, and wastewater were capable of hemolyzing horse erythrocytes. Vergis at al. showed in their study that 23 (11%) of 211 *E. faecalis* clinical isolates were able to produce hemolysin. Vancomycin resistance was detected in 2 (9%) of the 23 hemolysin-producing *E. faecalis* isolates [57]. Our sensitivity data show that 3 (17.65%) out of 17 hemolytic *E. faecalis* strains were VAN-R. The same result was observed for the CIP-R hemolytic *E. faecalis* strains. Conversely, cytolysin is not a characteristic virulence factor of *E. faecium*, and only 3.33% of isolates from food showed hemolytic activity.

This study is the first and largest Croatian study analyzing enterococci from various isolation sources based on virulence factors, including hydrophobicity index, biofilm formation ability, hemolysis, and susceptibility to antibiotics CIP and VAN, including susceptibility to biocides TCL and CHX across different isolation sources. The obtained results emphasize the importance of the One Health Concept in increasing AMR. Still, there were some limitations. First of all, this is a single-center study mainly having clinical isolates from one hospital site, so overall conclusions should be implemented with caution. Seasonal variations and sampling from routine clinical work could also influence results. Also, a larger sample could provide a statistically significant difference that might not be obtained in our study.

## 5. Conclusions

Antimicrobial resistance (AMR) is a global issue, with resistant organisms capable of spreading rapidly through healthcare facilities, food, and the environment. This study investigated the phenotypic susceptibility of *Enterococcus* isolates from various sources to antibiotics (vancomycin and ciprofloxacin) and biocides (triclosan and chlorhexidine) and examined the association of resistance with certain virulence factors. The results for *E. faecalis* and *E. faecium* revealed widespread phenotypic resistance to vancomycin and ciprofloxacin, irrespective of the isolation site. However, reduced sensitivity (tolerance) to biocides such as chlorhexidine and triclosan could not be demonstrated. Our findings indicate that the hydrophobicity of enterococcal cell surfaces does not correlate with biofilm-formation ability.

## Figures and Tables

**Table 1:** Association of *Enterococcus* spp. susceptibility to ciprofloxacin and vancomycin according to isolation sources.

			Clinical n (%)	Food n (%)	Wastewater n (%)	*p*-Value
*E. faecalis*	CIP	R	12 (54.55)	6 (27.27)	4 (18.18)	0.094
		S	17 (25.37)	24 (35.82)	26 (38.81)	0.368
	VAN	R	3 (27.27)	1 (9.09)	7 (63.64)	0.078
		S	26 (33.33)	29 (37.18)	23 (29.49)	0.707
*E. faecium*	CIP	R	30 (53.57)	11 (19.64)	15 (26.79)	0.005
		S	0 (0.0)	19 (55.88)	15 (44.12)	0.493
	VAN	R	25 (75.76)	2 (6.06)	6 (18.18)	<0.001
		S	5 (8.77)	28 (49.12)	24 (42.11)	<0.001

CIP = ciprofloxacin, VAN = vancomycin, R = resistant, S = susceptible.

**Table 2:** Distribution of TCL/CHX MIC_50_ and MIC_90_ (mg/L) according to isolation source.

		Triclosan (mg/L)		Chlorhexidine (mg/L)	
Species	Isolation Source	MIC_50_	MIC_90_	MIC_50_	MIC_90_
*E. faecalis*	Clinical specimens	8	8	16	32
	Food	8	16	4	8
	Wastewater	16	32	8	16
*E. faecium*	Clinical specimens	8	8	8	16
	Food	8	16	2	4
	Wastewater	4	8	2	4

**Table 3:** Biofilm-forming strength of enterococci isolated from different sources.

Biofilm Strength	*E. faecalis* n (%)			*E. faecium* n (%)		
	Food	Clinical Specimens	Wastewater	Food	Clinical Specimens	Wastewater
No biofilm (I)	0 (0.00)	0 (0.00)	2 (6.67)	4 (13.33)	1 (3.33)	20 (66.67)
Weak (II)	0 (0.00)	2 (6.67)	2 (6.67)	0 (0.00)	0 (0.00)	6 (20.00)
Moderate (III)	1 (3.45)	6 (20.00)	10 (33.33)	4 (13.33)	5 (16.67)	4 (13.33)
Strong (IV)	28 (96.55)	22 (73.33)	16 (53.33)	22 (73.33)	24 (80.00)	0 (0.00)
Total biofilm producers (II, III, IV)	29 (100.00)	30 (100.00)	28 (93.33)	26 (86.67)	29 (96.67)	10 (33.33)

**Table 4:** *E. faecalis*—association between biofilm-forming strength and CIP and VAN susceptibility.

Biofilm Strength	CIP-R n (%)	CIP-S n (%)	*p*-Value
No biofilm (I)	1 (50.00)	1 (50.00)	
Weak (II)	0 (0.00)	4 (100.00)	NA
Moderate (III)	5 (29.41)	12 (70.59)	0.009
Strong (IV)	16 (24.24)	50 (75.76)	<0.001
Total biofilm producers (II, III, IV)	21 (24.14)	66 (75.86)	<0.001
Biofilm strength	VAN-R n (%)	VAN-S n (%)	*p*-value
No biofilm (I)	0 (0.00)	2 (100.00)	
Weak (II)	0 (0.00)	4 (100.00)	NA
Moderate (III)	3 (17.65)	14 (82.35)	0.008
Strong (IV)	8 (12.12)	58 (87.88)	<0.001
Total biofilm producers (II, III, IV)	11 (12.64)	76 (87.36)	<0.001

CIP = ciprofloxacin, VAN = vancomycin, R = resistant, S = susceptible, NA = not applicable.

**Table 5:** *E. faecium*—association between biofilm-forming strength and CIP and VAN susceptibility.

Biofilm Strength	CIP-R n (%)	CIP-S n (%)	*p*-Value
No biofilm (I)	12 (48.00)	13 (52.00)	
Weak (II)	4 (66.67)	2 (33.33)	NA
Moderate (III)	9 (69.23)	4 (30.77)	0.166
Strong (IV)	31 (67.39)	15 (32.61)	0.018 *
Total biofilm producers (II, III, IV)	44 (67.69)	21 (32.31)	0.004 *
Biofilm strength	VAN-R n (%)	VAN-S n (%)	*p*-value
No biofilm (I)	8 (32.00)	17 (68.00)	
Weak (II)	2 (33.33)	4 (66.67)	NA
Moderate (III)	4 (30.77)	9 (69.23)	0.166
Strong (IV)	19 (41.30)	27 (58.70)	0.238
Total biofilm producers (II, III, IV)	25 (38.46)	40 (61.54)	0.063

CIP = ciprofloxacin, VAN = vancomycin, R = resistant, S = susceptible, NA = not applicable, * *p* < 0.05.

**Table 6:** Hemolytic activity of *E. faecalis* and *E. faecium* strains.

		*E. faecalis* n (%)			*E. faecium* n (%)		
	β-Hemolysis	Food	Clinical Specimens	Wastewater	Food	Clinical Specimens	Wastewater
Aerobic incubation	No	27 (90.00)	26 (89.66)	24 (80.00)	27 (90.00)	30 (100.00)	30 (100.00)
	Yes	3 (10.00)	3 (10.34)	6 (20.00)	3 (10.00)	0 (0.00)	0 (0.00)
	Total	30 (100.00)	29 (100.00)	30 (100.00)	30 (100.00)	30 (100.00)	30 (100.00)
Anaerobic incubation	No	26 (86.67)	23 (79.31)	23 (76.67)	27 (90.00)	30 (100.00)	30 (100.00)
	Yes	4 (13.33)	6 (20.69)	7 (23.33)	3 (10.00)	0 (0.00)	0 (0.00)
	Total	30 (100.00)	29 (100.00)	30 (100.00)	30 (100.00)	30 (100.00)	30 (100.00)

## Data Availability

The original contributions presented in the study are included in the article/Supplementary Material, further inquiries can be directed to the corresponding authors.

## Supplementary Materials

The following supporting information can be downloaded at: https://www.mdpi.com/article/10.3390/microorganisms12091808/s1, Table S1: *E. faecium* relationship between the MIC values for triclosan and chlorhexidine, and the results for vancomycin and ciprofloxacin; Table S2: *E. faecalis* relationship between the MIC values for triclosan and chlorhexidine, and the results for vancomycin and ciprofloxacin.
