# Supplementary material for: Virulence Factors and Susceptibility to Ciprofloxacin, Vancomycin, Triclosan, and Chlorhexidine among Enterococci from Clinical Specimens, Food, and Wastewater
Source: Microorganisms. 2024 Sep 1;12(9):1808. doi: 10.3390/microorganisms12091808 (PMC11434535; doi:10.3390/microorganisms12091808)
Supplement: Supplementary file 1 [file microorganisms-12-01808-s001.zip › microorganisms-3101765-supplementary.pdf]

**The relationship between the MIC values for triclosan and chlorhexidine and the results for vancomycin and ciprofloxacin is shown in Supplementary Table S1 for *E. faecium* and in Supplementary Table S2 for *E. faecalis*.**

**Supplementary Table S1.** *E. faecium* relationship between the MIC values for triclosan and chlorhexidine, and the results for vancomycin and ciprofloxacin

| *E. faecium* (n=90) | TCL $MIC_{50}$ mg/L | TCL $MIC_{90}$ mg/L | TCL range mg/L | CHX $MIC_{50}$ mg/L | CHX $MIC_{90}$ mg/L | CHX range mg/L |
|---|---|---|---|---|---|---|
| **CIP R (n=56)** | 4 | 16 | 2-16 | 4 | 16 | 2-32 |
| **CIP S (n=34)** | 8 | 16 | 4-32 | 2 | 4 | 1-8 |
| **VAN R (n=33)** | 4 | 8 | 2-8 | 4 | 16 | 2-32 |
| **VAN S (n=57)** | 8 | 16 | 4-32 | 2 | 4 | 1-16 |

**Supplementary Table S2.** *E. faecalis* relationship between the MIC values for triclosan and chlorhexidine, and the results for vancomycin and ciprofloxacin

| *E. faecalis* (n=89) | TCL $MIC_{50}$ mg/L | TCL $MIC_{90}$ mg/L | TCL range mg/L | CHX $MIC_{50}$ mg/L | CHX $MIC_{90}$ mg/L | CHX range mg/L |
|---|---|---|---|---|---|---|
| **CIP R (n=22)** | 8 | 16 | 4-32 | 8 | 16 | 2-64 |
| **CIP S (n=67)** | 8 | 16 | 4-64 | 8 | 16 | 2-64 |
| **VAN R (n=11)** | 8 | 16 | 4-64 | 8 | 8 | 4-16 |
| **VAN S (n=78)** | 8 | 16 | 4-64 | 8 | 16 | 2-64 |
